# Mutation patterns and evolutionary action score of *TP53* enable identification of a patient population with poor prognosis in advanced non‐small cell lung cancer

**DOI:** 10.1002/cam4.5447

**Published:** 2022-11-28

**Authors:** Wensheng Jiang, Huanqing Cheng, Lili Yu, Jie Zhang, Yihui Wang, Yun Liang, Feng Lou, Huina Wang, Shanbo Cao

**Affiliations:** ^1^ Department of Cardiothoracic Surgery Yantaishan Hospital Yantai China; ^2^ Department of Medicine Acornmed Biotechnology Co., Ltd. Beijing China

**Keywords:** EAp53, mutational landscape, NSCLC, overall survival, TP53

## Abstract

**Background:**

*TP53* mutations are frequent in non‐small cell lung cancer (NSCLC). Different categories of *TP53* mutations may be associated with survival in advanced NSCLC, but their effect on prognosis is diverse. To date, a comprehensive comparison of the relationship between different classes of *TP53* alterations and survival in advanced NSCLC has rarely been performed. Moreover, the prognostic significance of a novel approach called the evolutionary action of *TP53* (EAp53) in advanced NSCLC is unclear.

**Methods:**

A total of 210 patients with NSCLC harboring *TP53* mutation data were enrolled. Genomic and clinical data for the Memorial Sloan Kettering Cancer Center (MSKCC) cohort with advanced NSCLC were obtained from cBioPortal. Relationship between clinical characteristics and *TP53* mutations was performed by Fisher's exact test or χ^2^ test. Overall survival (OS) analysis was evaluated using Kaplan–Meier method and Cox proportional hazards regression model.

**Results:**

*TP53* mutations were identified in 51.4% of NSCLC patients and were mainly located in exons 5, 7, and 8. The distribution patterns of missense and truncating mutations of *TP53* were remarkably different. Among patients with advanced NSCLC who never received immune checkpoint inhibitor treatments, EAp53 high‐risk mutations were significantly associated with poor OS in both our cohort and the MSKCC cohort. Moreover, marked differences were observed in the mutational landscape between patients with EAp53 high‐risk mutations (HR group) and other patients (OT group). The HR group displayed higher mutation frequencies in the RTK, cell cycle, and DNA damage repair (DDR) pathways than the OT group. In addition, the tumor mutation burden in the HR group was significantly higher than that in the OT group.

**Conclusions:**

This study provided important insights into the molecular‐clinical profile of *TP53*‐mutated NSCLC patients. Moreover, the data revealed that EAp53 high‐risk mutations were an independent prognostic factor for worse OS in advanced NSCLC.

## Graphical abstract

This study provided important insights into the molecular‐clinical profile of TP53‐mutated NSCLC patients. The data revealed that EAp53 high‐risk mutations were an independent prognostic factor for worse OS in advanced NSCLC.

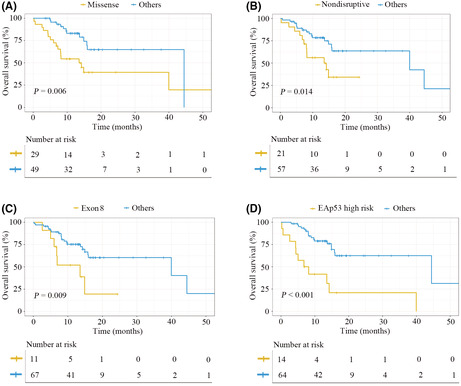

## 1 INTRODUCTION

Lung cancer is a leading cause of cancer‐associated mortality worldwide.^1^ The predominant histological type of lung cancer is non‐small cell lung cancer (NSCLC), which accounts for approximately 85% of all lung cancer patients.^2^ With the development of medical science, targeted therapies and immune checkpoint blockade (ICI) treatments remarkably improved overall survival in advanced NSCLC.^3^ Even so, more than 50% of patients with advanced NSCLC do not carry targetable driver alterations and cannot benefit from ICI treatments, and chemotherapy remains a crucial treatment strategy in this population.^4^, ^5^ Currently, although the majority of advanced NSCLC patients can benefit from targeted therapies and chemotherapies, the survival time has been shown to significantly vary among different patients. Therefore, it is of great importance to investigate the possible mechanisms related to the heterogeneous outcomes.

*TP53*, a tumor suppressor gene, is the most commonly mutated gene in various human malignancies, and mutations occur in approximately 50% of NSCLC patients.^6^, ^7^ The *TP53* gene encodes tumor protein p53, which is a transcription factor and plays crucial roles in DNA damage repair (DDR), cell cycle regulation, and apoptosis.^8^, ^9^ Previous reports revealed that the majority of *TP53* alterations in tumors were distributed in the DNA‐binding region (between amino acid residues 102–292), and the most frequently affected residues were located in the conserved 175, 248, 249, 273, and 282 sites.^10^, ^11^, ^12^ Numerous studies have indicated the prognostic role of *TP53* variants in advanced NSCLC, but most of them mainly focused on the effect of different *TP53* mutation categories in patients receiving targeted therapies. For instance, prior studies reported that *TP53* missense mutations, nondisruptive mutations, and exon 8 mutations acted as negative‐independent prognostic indicators in *EGFR‐*mutated NSCLC patients.^13^, ^14^, ^15^ However, limited studies have investigated the prognostic importance of different *TP53* mutation classes in advanced NSCLC patients treated with chemotherapies. Furthermore, to date, the prognostic value of different *TP53* mutation types among patients with advanced NSCLC regardless of targeted therapies and chemotherapies is largely unknown.

Most *TP53* alterations were found to be missense mutations, but they all were not functionally equivalent. Recently, a novel algorithm called the evolutionary action score of *TP53* (EAp53), which was used to categorize patients with*TP53* missense alterations into high‐risk and low‐risk groups, exhibited great prognostic value in colorectal cancer, head and neck cancer, and acute myeloid leukemia.^16^, ^17^, ^18^ However, the prognostic role of EAp53 in advanced NSCLC remains elusive. Herein, we conducted targeted sequencing in a large NSCLC cohort to uncover the mutation patterns of *TP53*. Moreover, in advanced NSCLC patients who did not receive ICI treatments, the association between different *TP53* mutation categories and prognosis was investigated and compared in our cohort to determine whether the EAp53 stratification system could act as a prognostic factor in advanced NSCLC. We further validated our findings in the MSKCC cohort. In addition, the molecular features associated with EAp53 classification were explored.

## 2 MATERIALS AND METHODS

### 2.1 Patients

This was a retrospective study that enrolled 210 patients with NSCLC who underwent next‐generation sequencing (NGS) of *TP53* from May 2014 to June 2018. All patient samples were continuously collected. The clinical stages of tumors ranged from I to IV and were verified according to the American Joint Committee on Cancer staging scheme (7th edition). None of the patients ever received ICI treatments. Genomic and clinical data for the MSKCC cohort with advanced NSCLC were downloaded from cBioPortal.^19^ In the MSKCC cohort, samples from 1,567 patients with advanced NSCLC were collected and sequenced. After excluding patients who received ICI treatments,^20^ a total of 1,189 patients with available OS data were included for subsequent analyses. Informed consent was obtained from all participants.

### 2.2 NGS

Sample DNA was extracted using the QIAamp Genomic DNA kit (Germany, QIAGEN). Then, sequencing libraries were constructed based on the manufacturer's instructions (Illumina Inc.). The libraries were enriched with a panel of the nine most common driver genes (*TP53*, *EGFR*, *KRAS*, *BRAF*, *ERBB2*, *ALK*, *ROS1*, *MET*, and *RET*) in NSCLC. The sequencing panel covered all the exons of *TP53* gene. The target‐enriched libraries were then pooled and sequenced on the NovaSeq6000 System (Illumina Inc.). After removing the low‐quality sequencing data, reads were aligned to the reference human genome (GRCh37) with Burrows‐Wheeler alignment tool v0.7.12. Base recalibration was carried out utilizing GATK software v3.8. The calling of single nucleotide variants and small insertions or deletions was performed by MuTect2 v1.1.7. Copy number variant calling was analyzed with CONTRA v2.0.8.

### 2.3 Classification of *TP53* mutations

Disruptive alterations, as described previously,^21^, ^22^ included inactivating mutations (nonsense, frameshift, and splice site mutations) or missense substitutions within the L2 or L3 loop of the DNA‐binding domain with codons of amino acids of a different polarity or charge group. All other variants were categorized as nondisruptive. Moreover, missense mutations were classified as EAp53 low‐risk (EAp53 score ≤ 75) or EAp53 high‐risk (EAp53 score > 75) types, which were analyzed by a novel algorithm according to evolutionary sensitivity and amino acid conservation.^23^ The EAp53 score is available from http://mammoth.bcm.tmc.edu/EAp53/.

### 2.4 Statistical analysis

GraphPad Prism (version 8.0.2) and R (version 4.1.3) were used to perform the statistical analyses. Differences in continuous variables were analyzed using the Mann–Whitney *U*‐test. Fisher's exact test or χ^2^ test was utilized to determine the relationship between clinical characteristics and genomic mutations. Survival analysis was conducted using the Kaplan–Meier method and log‐rank test, and multivariate analysis was carried out using a Cox proportional hazards regression model. Results with a two‐sided *p* < 0.05 were considered statistically significant.

## 3 RESULTS

### 3.1 Clinical characteristics of patients

In this study, a total of 210 patients diagnosed with NSCLC were enrolled. Among all the patients, the overall median age at diagnosis was 65 years (range, 34–87 years), 50.0% of the patients were smokers, 60.0% of the patients were male, and 77.1% of the patients were diagnosed with lung adenocarcinoma. The proportions of patients with stage I, II, III, and IV disease were 4.3%, 3.8%, 14.8%, and 75.2%, respectively. In stage IV patients, 32.3% presented with multiple organ metastases. The clinical characteristics of the patients are listed in Table 1.

**TABLE 1:** Clinical characteristics of 210 patients with NSCLC

Clinical characteristics	Number (%)
Age, years, median (range)	65 (34–87)
Sex	
Male	126 (60.0%)
Female	84 (40.0%)
Smoking status	
Smoker	105 (50.0%)
Nonsmoker	105 (50.0%)
Pathology	
LUAD	162 (77.1%)
LUSC	46 (21.9%)
ASC	2 (1.0%)
Clinical Stage	
I	9 (4.3%)
II	8 (3.8%)
III	31 (14.8%)
IV	158 (75.2%)
Unknown	4 (1.9%)
Multiple organ metastasis (stage IV)	
Yes	51 (32.3%)
No	105 (66.5%)
Unknown	2 (1.3%)

Abbreviations: ASC, adenosquamous carcinoma; LUAD, lung adenocarcinoma; LUSC, lung squamous cell carcinoma.

### 3.2 Mutation patterns of *TP53*

**FIGURE 1:**
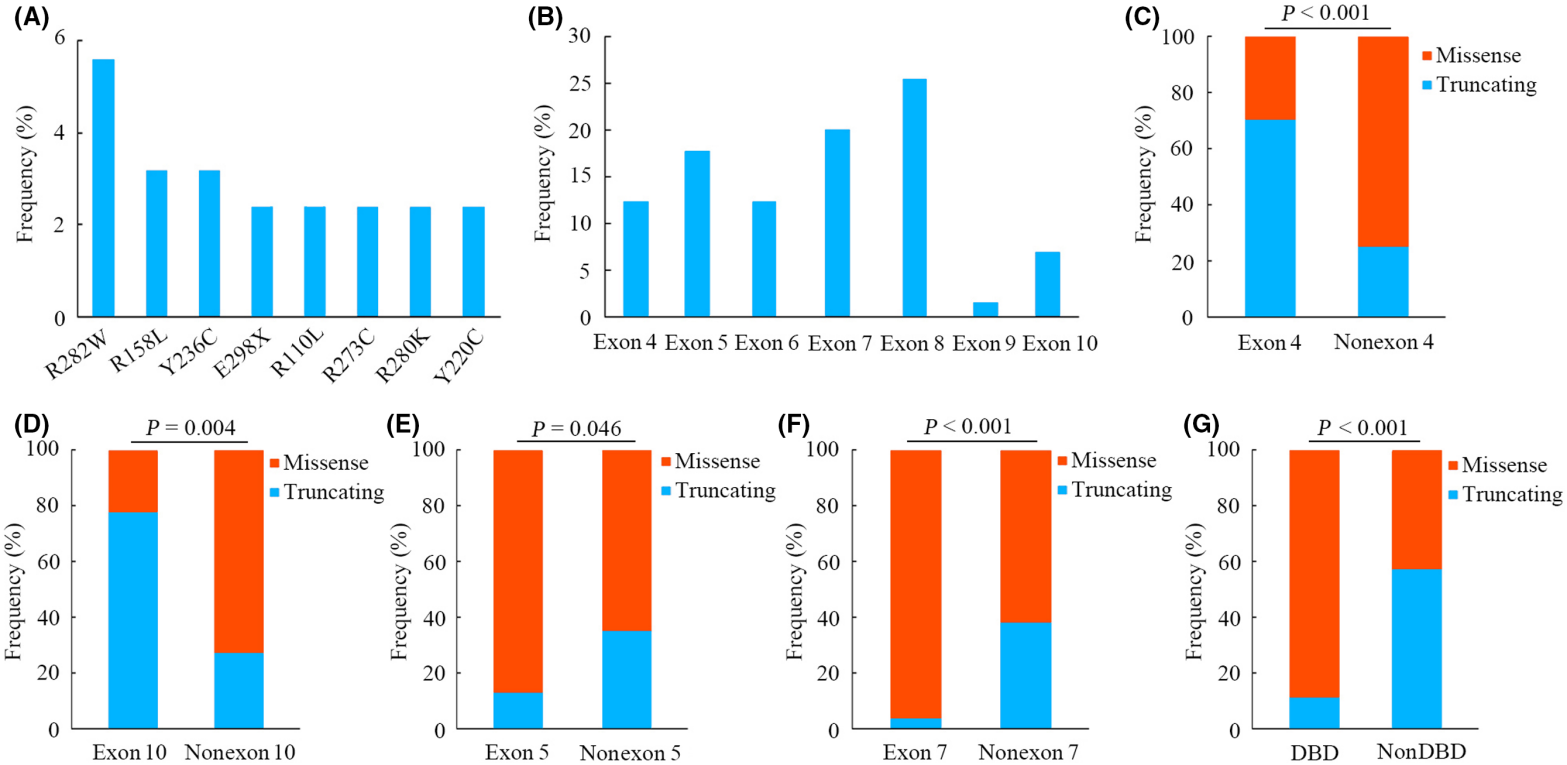
Distribution patterns of *TP53* mutations in patients with non‐small cell lung cancer (NSCLC). (A) Hot‐spot mutations of *TP53* in NSCLC. The frequency of each mutation is more than 2%. (B) The exon location of all *TP53* mutations. (C‐G) Distribution status of missense and truncating mutations of *TP53* in exon 4 (C), exon 10 (D), exon 5 (E), exon 7 (F), and DBD (G). DBD, DNA‐binding domain

Overall, a total of 125 *TP53* alterations were detected in 108 patients (51.4%). The frequency of *TP53* mutations was markedly associated with male sex and smoker status (*p* < 0.001 and *p* = 0.013, respectively) (Figure S1A,B). No significant difference was observed in the *TP53* mutation rate between younger patients (age ≤65 years) and older patients (age >65 years) (*p* = 0.581) (Figure S1C). In metastatic tumors, the prevalence of *TP53* mutations was significantly different in patients with single‐ and multiple‐organ metastases (*p* = 0.036) (Figure S1D). Among all *TP53* mutations, the most common variants were *R282W*, *R158L*, *Y236C*, *E298X*, *R110L*, *R273C*, *R280K*, and *Y220C* (Figure 1A). *TP53* mutations were mainly located in exons 5, 7, and 8 (Figure 1B), and 30.9% of them were truncating mutations (nonsense, splice site, and frameshift mutations). The frequencies of truncating and missense mutations were further compared among different exons, which showed that the frequency of truncating mutations was remarkably higher than that of missense mutations in exons 4 and 10 (*p* < 0.001 and *p* = 0.004, respectively) (Figure 1C,D). However, the opposite phenomenon was observed in exons 5 and 7 (*p* = 0.046 and *p* < 0.001, respectively) (Figure 1E,F). Since *TP53* is a transcription factor, we further analyzed the association between mutation types and different domains. Notably, the majority of truncating mutations were distributed in the non‐DNA‐binding domain, whereas missense mutations were mainly located in the DNA‐binding domain (*p* < 0.001) (Figure 1G).

### 3.3 Association of different *TP53* mutation types with survival in the study cohort

**FIGURE 2:**
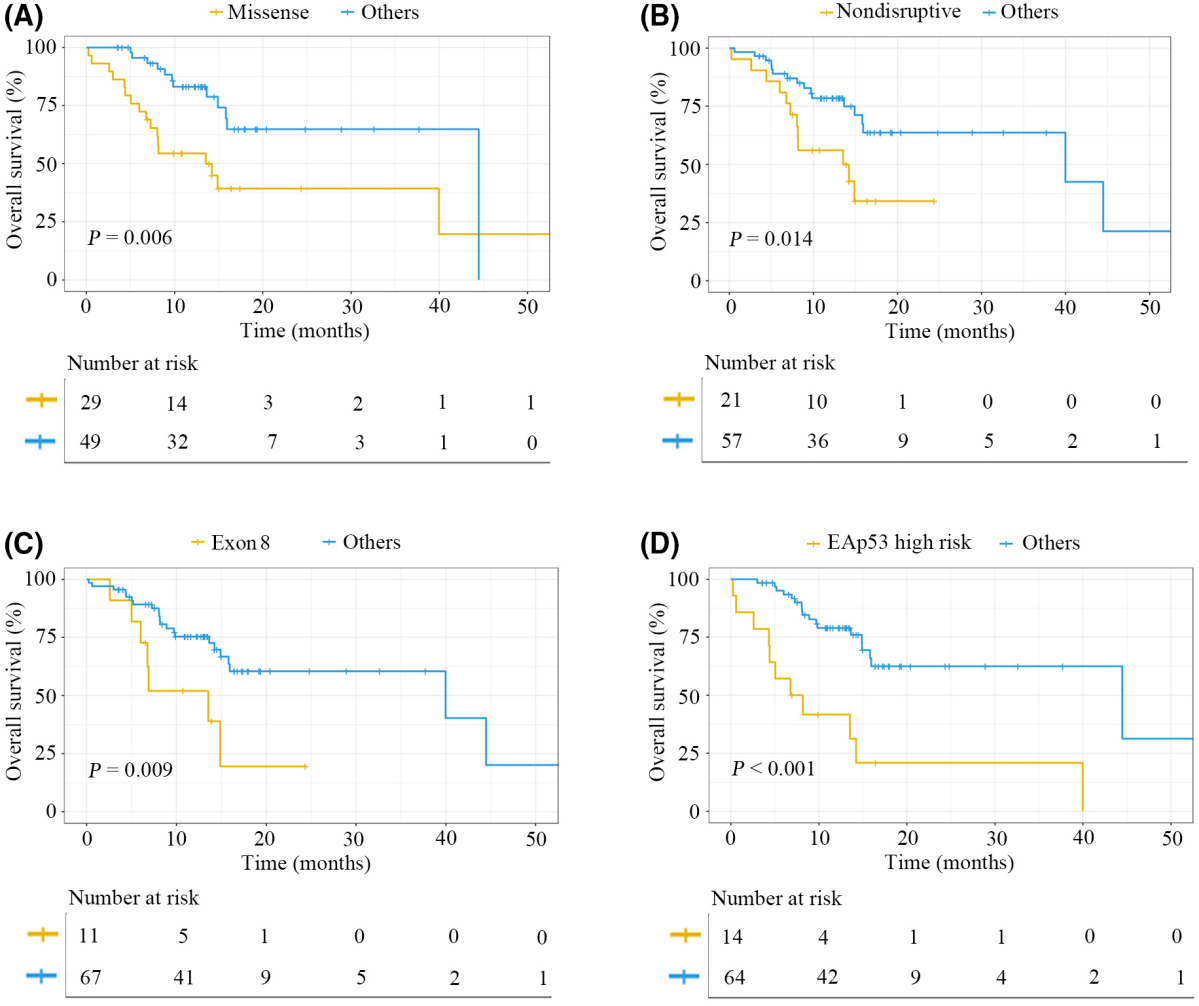
Kaplan–Meier estimates of OS for advanced NSCLC patients in our cohort stratified by different classes of *TP53* alterations

Most of the enrolled patients (90.0%) had advanced (stage III/IV) tumors. Among this population, OS data were collected from 78 patients who never received ICI treatments, and the prognostic significance of different *TP53* mutation classes was evaluated. Based on previous studies, we classified *TP53* variants into missense/truncating, exon 8/nonexon 8, and nondisruptive/disruptive mutation types. First, the association between missense mutations of *TP53* and prognosis was conducted, which showed that missense mutations were inversely correlated with OS (*p* = 0.023) (Figure S2A). No OS difference was found between patients with *TP53* truncating mutations and patients with *TP53* WT (*p* = 0.570) (Figure S2B), and then these patients were grouped together into a single category. Further analysis demonstrated that patients with *TP53* missense mutations had a worse prognosis than other patients (*p* = 0.006) (Figure 2A). Next, a comparison of prognosis between patients with disruptive and nondisruptive mutations of *TP53* was performed, and we observed that nondisruptive *TP53* mutations were related to poor OS (*p* = 0.028) (Figure S3A). No significant difference was observed in OS between patients with disruptive *TP53* mutations and those with *TP53* WT (*p* = 0.251) (Figure S3B), and subsequently, these patients were combined into a single group. It was observed that patients carrying *TP53* nondisruptive mutations exhibited worse OS than other patients (*p* = 0.014) (Figure 2B). Moreover, the relationship of *TP53* mutations in exon 8 with survival time was further explored. *TP53* alterations occurring on exon 8 were found to correspond with a dismal prognosis (*p* = 0.012) (Figure S4A). There was no OS difference between patients with *TP53* nonexon 8 mutations and *TP53* WT (*p* = 0.106) (Figure S4B), and we then combined these patients into a single class. It was further found that patients harboring *TP53* mutations in exon 8 had shorter OS when compared to other patients (*p* = 0.009) (Figure 2C). However, after multivariable adjustment, missense mutations, nondisruptive mutations, and exon 8 mutations of *TP53* were not independent prognostic factors for OS (Table 2).

**TABLE 2:** Univariate and multivariate analyses of factors associated with overall survival in our cohort

Characteristic	Parameter	Univariate analysis			Multivariate analysis		
		HR	95% CI	*p* value	HR	95% CI	*p* value
Age	>65 versus ≤65	0.85	0.40–1.77	0.660	0.82	0.34–1.95	0.650
Sex	Male versus Female	2.23	1.09–4.70	0.042	2.06	0.49–8.69	0.326
Smoking status	Smoker versus Nonsmoker	2.10	1.01–4.34	0.047	0.65	0.17–2.48	0.529
*EGFR* mutation status	MUT versus WT	0.25	0.12–0.52	<0.001	0.30	0.10–0.88	0.029
*TP53* mutation status	Missense versus Others	2.66	1.24–5.71	0.006	0.85	0.20–3.53	0.819
*TP53* mutation status	Nondisruptive	2.42	1.01–5.78	0.014	0.94	0.26–3.44	0.930
	versus. Others						
*TP53* mutation status	Exon 8 vs.	2.88	0.85–9.72	0.009		0.61–5.67	
	Others				1.87		0.272
*TP53* mutation status	EAp53 high‐risk	4.35	1.44–13.18	<0.001	3.63	1.14–11.62	0.030
	vs. Others						

Abbreviations: MUT, mutation; WT, wild type.

In addition, the correlation of EAp53 classification with survival in advanced NSCLC was further investigated. In the present study, all patients were categorized into four groups based on whether they possessed EAp53 high‐risk mutations, EAp53 low‐risk mutations, *TP53* truncating mutations, and *TP53* WT. Among the four groups, the high‐risk EAp53 group showed the worst OS (*p* < 0.001) (Figure S5A). No survival difference was found among the other three groups (*p* = 0.655) (Figure S5B). Further analysis demonstrated that patients carrying EAp53 high‐risk mutations exhibited strikingly worse OS than the other patients (*p* < 0.001) (Figure 2D). Moreover, after multivariable adjustment, EAp53 high‐risk mutations remained an independent negative prognostic factor for OS in advanced NSCLC (*p* = 0.030) (Table 2).

### 3.4 Validation of the prognostic role of EAp53 classification in the MSKCC cohort

**FIGURE 3:**
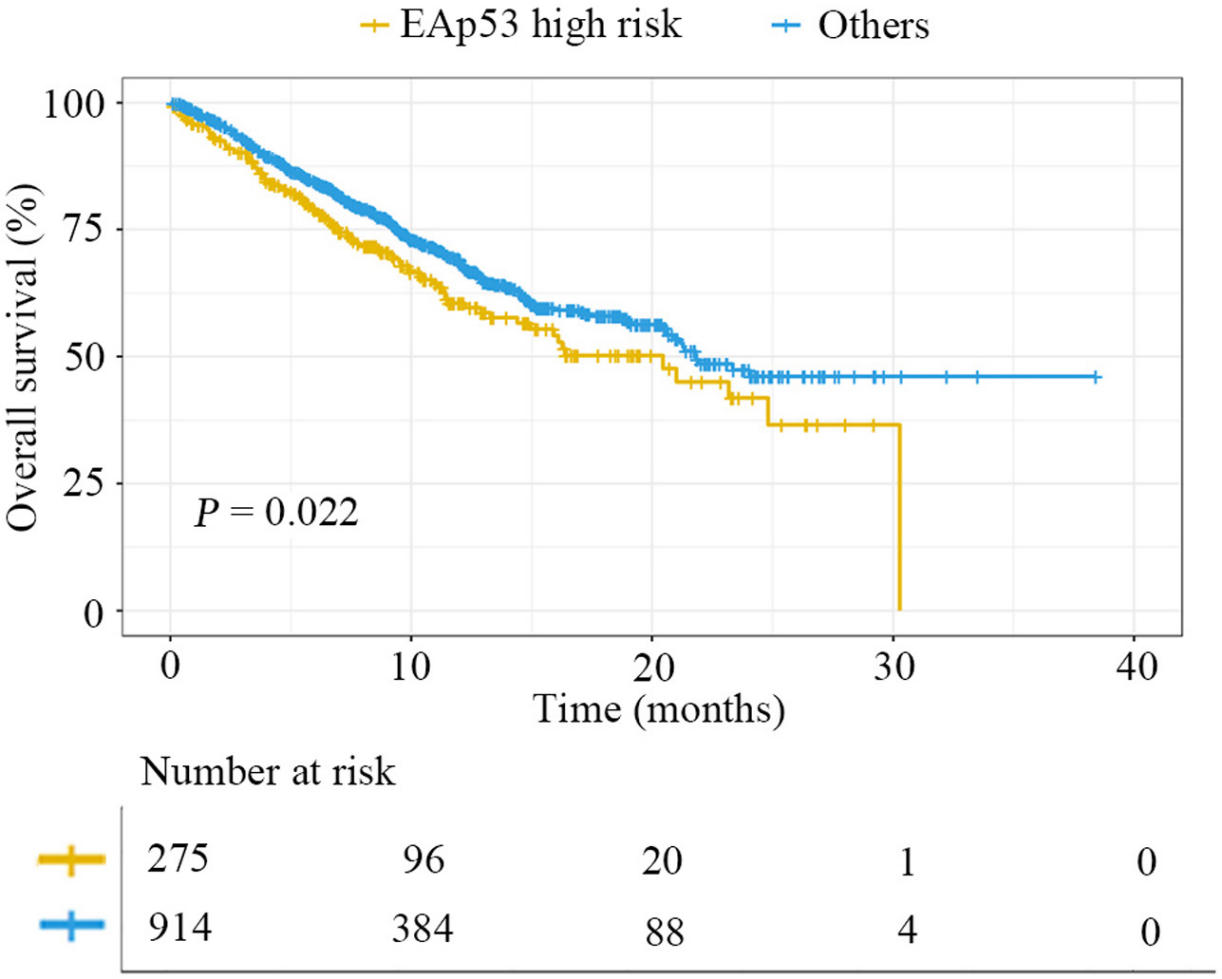
Association between OS and EAp53 classification in advanced NSCLC of the MSKCC cohort

In the MSKCC cohort, the association between different EAp53 mutation categories and OS was further validated. Among all the patients with advanced NSCLC, a total of 1,189 patients who harbored available OS data and never received ICI treatments were included for subsequent analyses. The results showed that patients with EAP53 high‐risk mutations were associated with significantly worse OS than the other patients (*p* = 0.022) (Figure 3). Furthermore, multivariate analysis showed that EAp53 high‐risk mutations remained an independent negative prognostic factor for OS in advanced NSCLC (*p* = 0.025) (Table S1).

### 3.5 Correlation between molecular features and EAp53 classification

**FIGURE 4:**
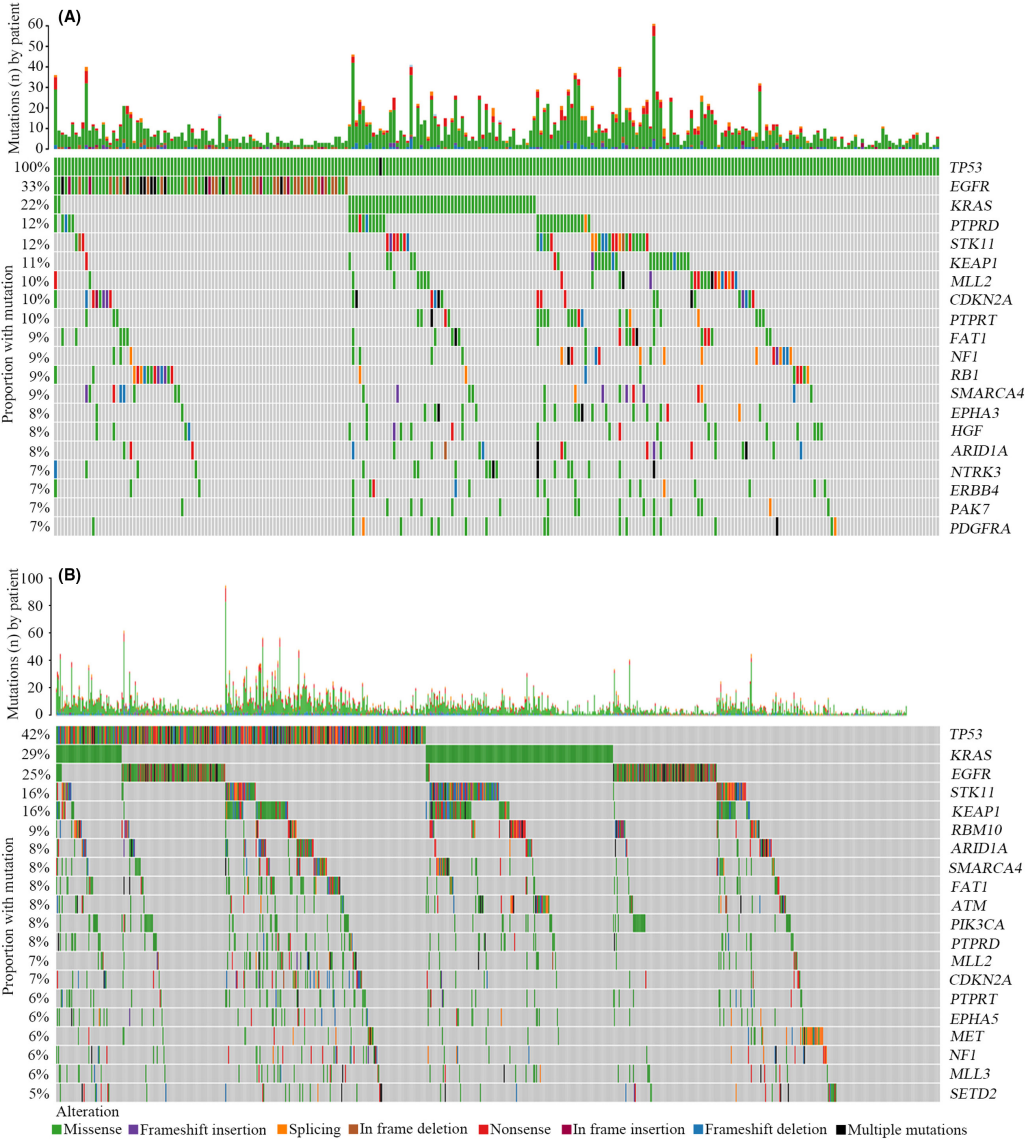
Genomic profiling of patients carrying EAp53 high‐risk mutations and other patients with advanced NSCLC. (A) Mutational landscape in patients with EAp53 high‐risk mutations. (B) Mutational landscape in patients without EAp53 high‐risk mutations

**FIGURE 5:**
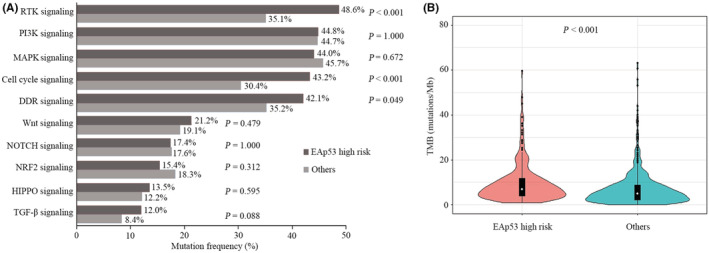
Comparison of the frequencies of genomic mutations in signaling pathways and tumor mutation burden (TMB) between patients harboring EAp53 high‐risk mutations and other patients. (A) Analysis of the prevalence of genomic mutations in signaling pathways between the two groups. (B) Comparison of the TMB between the two groups

To further explore the molecular characteristics related to patients carrying EAp53 high‐risk mutations, we compared the genomic profiles between patients with EAp53 high‐risk mutations (HR group) and other patients (OT group). Notably, the mutational landscape showed remarkable differences between the two groups. In the HR group, the most frequently mutated genes were *EGFR* (33%), *KRAS* (22%), *PTPRD* (12%), *STK11* (12%), *KEAP1* (11%), *MLL2* (10%), *CDKN2A* (10%), and *PTPRT* (10%) (Figure 4A). In the OT group, the most commonly mutated genes were *KRAS* (29%), *EGFR* (25%), *STK11* (16%), *KEAP1* (16%), and *RBM10* (9%) (Figure 4B). Although *EGFR*, *KRAS*, and *STK11* were the most frequently altered genes in both groups, their ranking orders and mutation frequencies were diverse. Statistical analysis revealed that the mutation frequencies of many genes (*EGFR*, *KRAS*, *PTPRD*, *PTPRT*, *RB1*, *STAG2*, *B2M*, etc.) between the two groups were significantly different (*p* < 0.05) (Figure S6). In addition, the altered signaling pathways between the two groups were further explored. We found that the frequencies of mutations involved in RTK, cell cycle, and DDR pathways in the HR group were strikingly higher than those in the OT group (Figure 5A). Further analysis demonstrated that the tumor mutation burden (TMB) in the HR group was significantly higher than that in the OT group (Figure 5B).

## 4 DISCUSSION

The present study comprehensively revealed the clinic‐molecular characteristics and prognostic value of *TP53* mutations in NSCLC. Herein, we found that *TP53* mutations frequently occurred in patients who smoked and male patients, which was consistent with previous reports on NSCLC.^24^, ^25^ Notably, the rate of *TP53* mutations was remarkably associated with multiple organ metastases in stage IV patients, implying that *TP53* variants confer poor prognosis in advanced NSCLC. Intriguingly, among the five hotspot codons (codons 175, 248, 249, 273, and 282) of *TP53* alterations in multiple types of cancer, only mutations in two codons (codons 273 and 282) were observed to be commonly altered in the study. Although *TP53* mutations frequently occur in human cancers, our results indicated distinctive patterns of *TP53* alterations in different malignancies. Missense mutations are the major type of *TP53* alterations and are mainly localized in the DNA‐binding domain.^7^ However, limited work has investigated the characteristics of *TP53* truncating mutations in NSCLC. Here, we found that 30.9% of *TP53* variants were truncating mutations that were mainly distributed in the non‐DNA‐binding domain and frequently occurred in exons 4 and 10. These results demonstrated the difference in distributions between missense and truncating mutations of *TP53*, further indicating the complexity of *TP53* variants in NSCLC.

High‐throughput sequencing technologies have enabled the elucidation of the molecular mechanisms associated with heterogeneous outcomes in advanced NSCLC. To date, an increasing number of studies have classified *TP53* alterations according to the differential biologic impacts caused by different mutant proteins, and their role in prognosis has been further explored. Among the categorizing methods, most were generally based on the functional impact and location of *TP53* mutations. In advanced NSCLC, a series of reports have discovered inferior OS in the presence of specific *TP53* variants, including missense mutations, nondisruptive mutations, or mutations existing in certain exons.^13^, ^14^, ^15^ Unfortunately, these studies generally focused on a particular group, such as patients receiving targeted therapies. However, among patients with advanced NSCLC, regardless of targeted therapies and chemotherapies, the prognostic role of different categories of *TP53* mutations is unclear. Recently, a novel algorithm termed EAp53 was used to stratify patients with*TP53* alterations into the high‐risk and low‐risk groups, and patients carrying high‐risk alterations were associated with lower survival in many types of cancer.^16^, ^17^ For instance, Osman et al. reported that EAp53 could be used to predict response and survival benefit in a subset of head and neck cancer patients treated with platinum‐based chemotherapy.^17^ Gleber‐Netto et al. indicated that EAp53 high‐risk mutations were correlated with an increased risk of extranodal extension in advanced oral squamous cell carcinoma.^26^ In addition, in patients with colorectal liver metastases, EAp53 high‐risk mutations were significantly associated with poor prognosis.^16^ All these findings highlighted the prognostic value of the EAp53 classification system across different tumor types. However, to date, no reports have investigated the applicability of EAp53 in the prediction of prognosis in advanced NSCLC.

Herein, we comprehensively explored and compared the relationship between different *TP53* mutation types and OS in patients with advanced NSCLC regardless of whether they received targeted therapies and chemotherapies. In univariate analysis, nondisruptive mutations, missense mutations, exon 8 mutations, and EAp53 high‐risk mutations were all significantly associated with inferior OS. However, after multivariate analysis, only EAp53 high‐risk mutations were an independent negative prognostic factor for OS. The results were further validated in an independent MSKCC cohort. *TP53* is the most frequently mutated gene in NSCLC, and most of the mutations identified are missense mutations. Currently, little is known regarding the functional effects of these missense mutations, and whether these mutations exhibit the same prognostic role is questionable. In this study, we observed that the EAp53 classification system, which was analyzed according to a genotype–phenotype model via an evolutionary function evaluation,^16^ could effectively stratify *TP53* missense mutations into high‐ and low = risk types and, for the first time, found the great prognostic value of this system in patients with advanced NSCLC. Compared to other classification system, EAp53 scores might currently be the most powerful prognostication tool for categorizing patients harboring *TP53* mutations in advanced NSCLC regardless of targeted therapies and chemotherapies.

The potential molecular features associated with poor prognosis in patients carrying EAp53 high‐risk mutations were further investigated. The present study revealed remarkable differences in the frequencies of multiple gene mutations between the HR and OT groups, demonstrating a specific mutational spectrum in the HR group. The DDR system plays an important role in maintaining genomic stability.^27^ Genomic instability is one of the hallmarks of cancer, playing an important role in tumorigenesis as well as tumor progression.^28^ Our findings demonstrated that the portion of DDR gene alterations in the HR group was significantly higher than that in the OT group, which implied that the HR group might have a greater degree of genomic instability resulting from deficiency of the mismatch repair of both DNA double‐strand breaks and single‐strand breaks, and chromosome maintenance. A prior study in metastatic breast cancer showed that DDR gene mutations were significantly correlated with high mutant‐allele tumor heterogeneity,^29^ further suggesting an association between DDR alterations and heterogeneity, and prognosis. Moreover, a number of studies have revealed that patients carrying DDR mutations exhibit high TMB and could benefit from PD‐1/PD‐L1 blockade in advanced NSCLC.^30^, ^31^ Consistently, TMB in the HR group was significantly higher than that in the OT group in our study. Since the patients in this study never received ICI treatments, our findings suggested that ICI treatments might be crucial treatment strategies in the HR group. It is well known that abnormalities in the cell cycle can cause uncontrolled cellular proliferation, which is significantly associated with a poor prognosis in multiple cancers.^32^, ^33^ In this study, we found that the rate of cell cycle pathway‐related gene mutations was remarkably higher in the HR group than in the OT group, further suggesting an inferior OS in the HR group.

The novelty of our study lies in the first finding of great prognostic role of EAp53 classification system in advanced NSCLC which might presently be the most powerful prognostication tool for classifying patients with *TP53* mutations whether they received targeted therapies or chemotherapies, as well as elucidating the molecular features related to this system. In conclusion, the present study investigated the distribution pattern of *TP53* mutations in a large NSCLC cohort, which is of great value in understanding the population and molecular characteristics of *TP53‐*mutated NSCLC patients. This study further comprehensively compared the associations between different classification systems of *TP53* mutations and prognosis in advanced NSCLC, demonstrating that the EAp53 stratification system could act as an independent prognostic factor for OS. Moreover, we discovered the specific genomic signatures of NSCLC patients with EAp53 high‐risk mutations. These results suggest that EAp53 high‐risk mutation subtypes should be taken into consideration in future personalized treatments and follow‐up strategies in advanced NSCLC.

## AUTHOR CONTRIBUTIONS

**Wensheng Jiang:** Formal analysis (lead); writing – original draft (lead); writing – review and editing (equal). **Huanqing Cheng:** Formal analysis (lead); writing – original draft (lead); writing – review and editing (equal). **Lili Yu:** Resources (equal); supervision (equal); writing – review and editing (equal). **Jie Zhang:** Resources (equal); supervision (equal); writing – review and editing (equal). **Yihui Wang:** Resources (equal); supervision (equal); writing – review and editing (equal). **Yun Liang:** Resources (equal); supervision (equal); writing – review and editing (equal). **Feng Lou:** Resources (equal); supervision (equal); writing – review and editing (equal). **Huina Wang:** Project administration (lead); supervision (equal); writing – review and editing (lead). **Shanbo Cao:** Project administration (lead); supervision (equal); writing – review and editing (lead).

## CONFLICT OF INTEREST

H.Q.C, H.N.W, F.L, and S.B.C are employees of Acornmed Biotechnology Co., Ltd. The other authors declare no conflict of interest.

## ETHICAL APPROVAL STATEMENT

The Ethical Committee of Yantaishan Hospital reviewed and approved this study (approval number: 2022055).

## Supporting information


Data S1

## Data Availability

The data that support the findings of this study are available from the corresponding author upon reasonable request.
