# Supplementary material for: Mutation patterns and evolutionary action score of TP53 enable identification of a patient population with poor prognosis in advanced non‐small cell lung cancer
Source: Cancer Med. 2022 Nov 28;12(6):6649–58. doi: 10.1002/cam4.5447 (PMC10067094; doi:10.1002/cam4.5447)
Supplement: Supplementary file 1 — Data S1 [file CAM4-12-6649-s001.docx]

**Supplementary Figures and Tables**

**
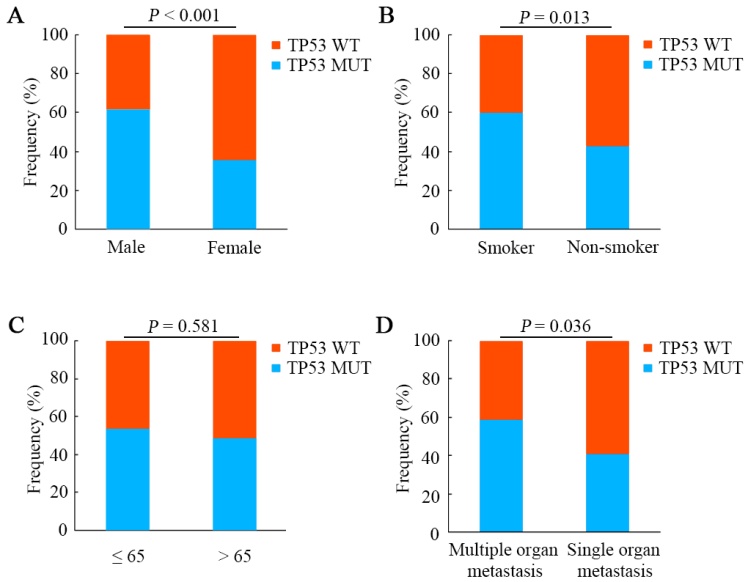
**

**Figure S1.** Association analyses between *TP53* mutations and different clinical characteristics. A-D, the relationship between *TP53* alterations and sex (A), smoking status (B), age (C), and organ metastasis patterns (D). Abbreviations: WT, wild type; MUT, mutation.

**
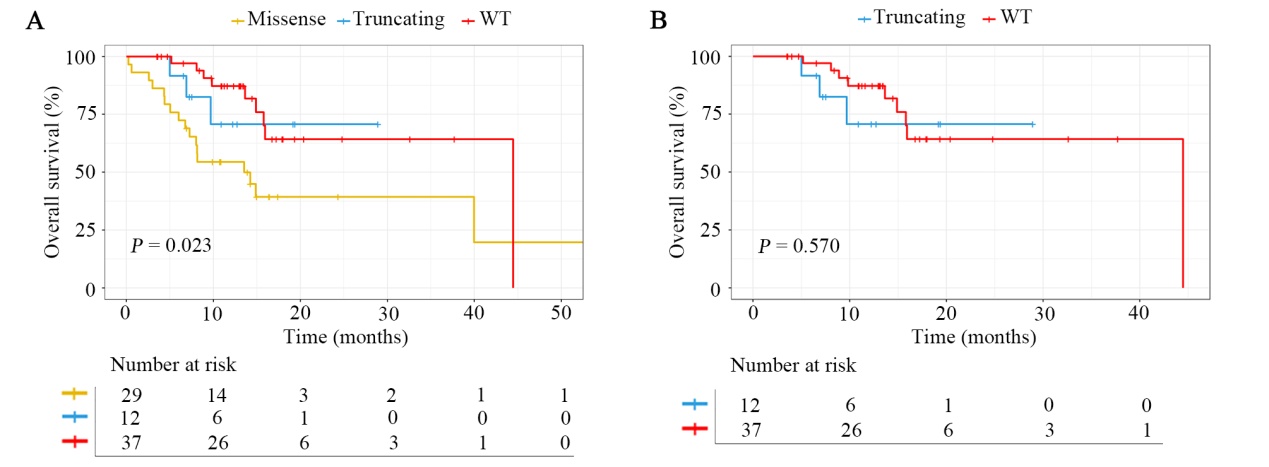
**

**Figure S2.** Effects of missense mutations of *TP53* on overall survival (OS) in advanced NSCLC. A, Kaplan-Meier estimates of OS for patients with 3 groups of *TP53* missense mutations, *TP53* truncating mutations, and *TP53* WT. B, Kaplan-Meier estimates of OS for patients with 2 groups of *TP53* truncating mutations and *TP53* WT.

**
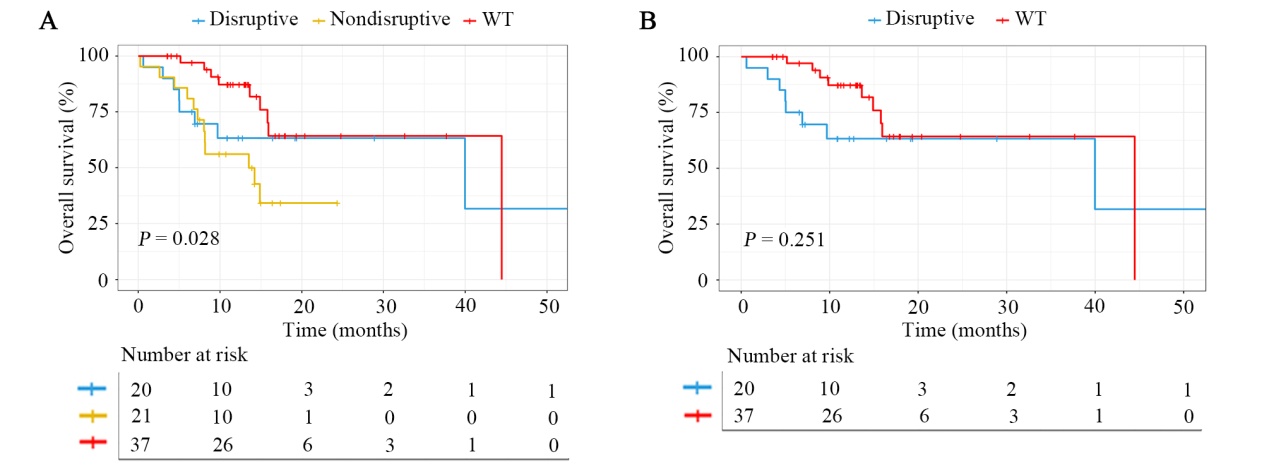
**

**Figure S3.** Effects of nondisruptive mutations of *TP53* on OS in advanced NSCLC. A, Kaplan-Meier estimates of OS for patients with 3 groups of *TP53* disruptive mutations, *TP53* nondisruptive mutations, and *TP53* WT. B, Kaplan-Meier estimates of OS for patients with 2 groups of *TP53* disruptive mutations and *TP53* WT.

**
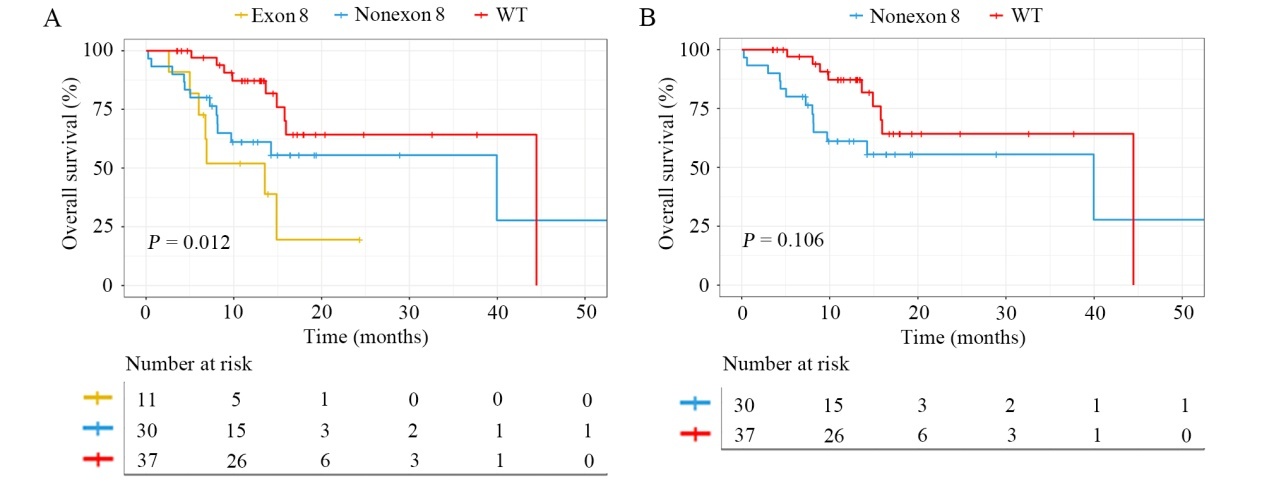
**

**Figure S4.** Effects of exon 8 mutations of *TP53* on OS in advanced NSCLC. A, Kaplan-Meier estimates of OS for patients with 3 groups of *TP53* exon 8 mutations, *TP53* nonexon 8 mutations, and *TP53* WT. B, Kaplan-Meier estimates of OS for patients with 2 groups of *TP53* nonexon 8 mutations and *TP53* WT.

**
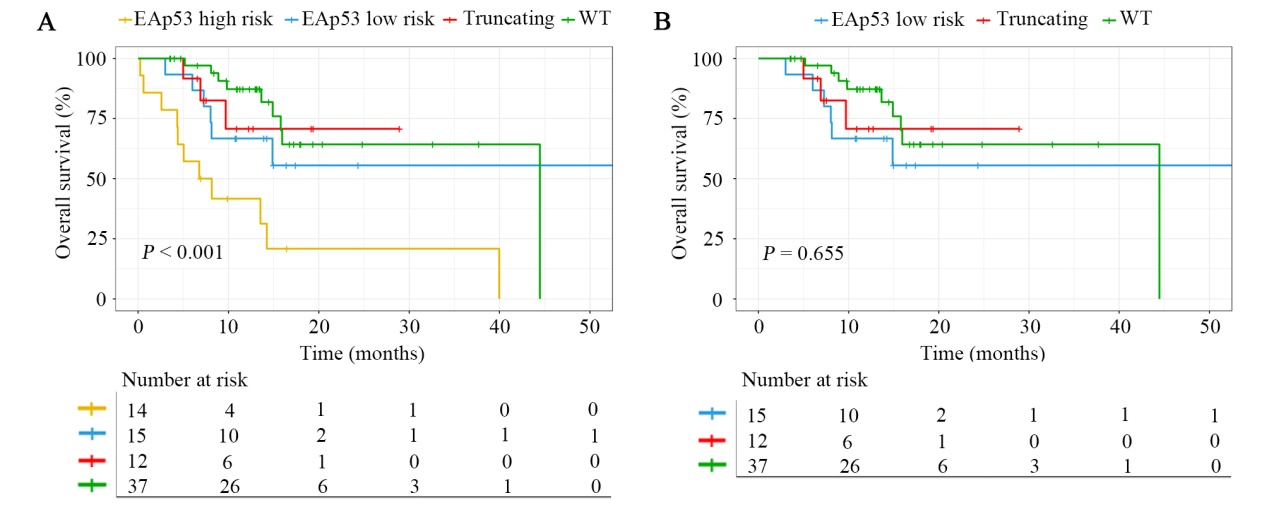
**

**Figure S5.** Effects of EAp53 classification on OS in advanced NSCLC. A, Kaplan-Meier estimates of OS for patients with 4 groups of EAp53 high risk mutations, EAp53 low risk mutations, *TP53* truncating mutations, and *TP53* WT. B, Kaplan-Meier estimates of OS for patients with 3 groups of EAp53 low risk mutations, *TP53* truncating mutations, and *TP53* WT.

**
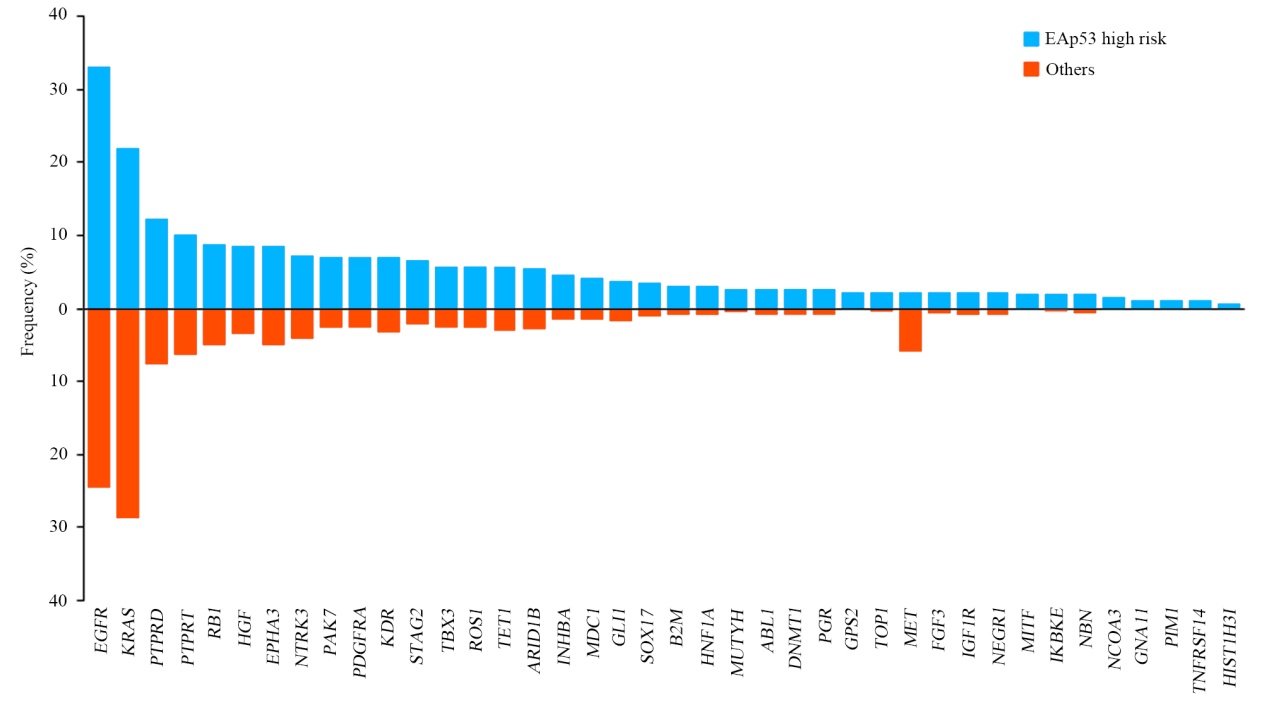
**

**Figure S6.** Comparison of the frequency of genomic mutations between patients harboring EAp53 high risk mutations and other patients.

**Table S1.** Univariate and multivariate analysis of EAp53 classification system associated with overall survival in MSKCC cohort.

| Characteristics | Parameters | Univariate analysis | | |  | Multivariate analysis | | | |
| --- | --- | --- | --- | --- | --- | --- | --- | --- | --- |
|  |  | HR | 95% CI | *P* value |  | HR | 95% CI | *P* value | |
| Sex | Male vs. Female | 1.43 | 1.16-1.76 | < 0.001 |  | 1.38 | 1.11-1.72 | | 0.004 |
| Smoking status | Smoker vs. Nonsmoker | 1.58 | 1.25-1.99 | < 0.001 |  | 1.35 | 1.03-1.77 | | 0.031 |
| *EGFR* mutation status | MUT vs. WT | 0.61 | 0.49-0.76 | < 0.001 |  | 0.69 | 0.52-0.90 | | 0.007 |
| *TP53* mutation status | EAp53 high risk vs. Others | 1.31 | 1.02-1.68 | 0.022 |  | 1.33 | 1.04-1.70 | | 0.025 |
